# Perfusion-weighted imaging in vestibular schwannoma: the influence that cystic status and tumor size have on perfusion profiles

**DOI:** 10.1590/0100-3984.2022.0035

**Published:** 2023

**Authors:** Felipe Constanzo, Bernardo Corrêa de Almeida Teixeira, Patricia Sens, Hamzah Smaili, Dante Luiz Escuissato, Ricardo Ramina

**Affiliations:** 1 Clínica Biobío, Concepción, Chile; 2 Hospital Clínico Regional de Concepción, Concepción, Chile; 3 Instituto de Neurologia de Curitiba (INC), Curitiba, PR, Brazil; 4 Universidade Federal do Paraná (UFPR), Curitiba, PR, Brazil

**Keywords:** Neuroma, acoustic, Magnetic resonance imaging/methods, Image enhancement/methods, Skull base neoplasms/ physiopathology, Neuroma acústico, Imageamento por ressonância magnética/métodos, Aumento da imagem/métodos, Neoplasias da base do crânio/fisiopatologia

## Abstract

**Objective:**

The perfusion profile of vestibular schwannomas (VSs) and the factors that influence it have yet to be determined.

**Materials and Methods:**

Twenty patients with sporadic VS were analyzed by calculating parameters related to the extravascular extracellular space (EES)—the volume transfer constant between a vessel and the EES (Ktrans); the EES volume per unit of tissue volume (Ve); and the rate transfer constant between EES and blood plasma (Kep)—as well as the relative cerebral blood volume (rCBV), and by correlating those parameters with the size of the tumor and its structure (solid, cystic, or heterogeneous).

**Results:**

Although Ktrans, Ve, and Kep were measurable in all tumors, rCBV was measurable only in large tumors. We detected a positive correlation between Ktrans and rCBV (r = 0.62, *p* = 0.031), a negative correlation between Ve and Kep (r = –0.51, *p* = 0.021), and a positive correlation between Ktrans and Ve only in solid VSs (r = 0.64, *p* = 0.048). Comparing the means for small and large VSs, we found that the former showed lower Ktrans (0.13 vs. 0.029, *p* < 0.001), higher Kep (0.68 vs. 0.46, *p* = 0.037), and lower Ve (0.45 vs. 0.83, *p* < 0.001). The mean Ktrans was lower in the cystic portions of cystic VSs than in their solid portions (0.14 vs. 0.32, *p* < 0.001), as was the mean Ve (0.37 vs. 0.78, *p* < 0.001). There were positive correlations between the solid and cystic portions for Ktrans (r = 0.71, *p* = 0.048) and Kep (r = 0.74, *p* = 0.037).

**Conclusion:**

In VS, tumor size appears to be consistently associated with perfusion values. In cystic VS, the cystic portions seem to have lower Ktrans and Ve than do the solid portions.

## Abstract (pt)

**Objetivo:**

O perfil de perfusão do schwannoma vestibular (SV) não tem sido estudado, nem os fatores que o influenciam.

**Materiais e Métodos:**

Vinte pacientes com SV esporádico foram analisados usando Ktrans, Ve, Kep e rCBV e correlacionados com tamanho e estádio cístico.

**Resultados:**

Ktrans, Ve e Kep foram medidos em todos os casos. rCBV só foi possível em tumores grandes. Ktrans e rCBV estavam correlacionados positivamente (r = 0,62, *p* = 0, 0 31 ) . Ve e Kep estavam negativamente correlacionados (r = –0,51, *p* = 0,021). Ktrans estava correlacionado positivamente com Ve em SVs sólidos (r = 0,64, *p* = 0,048). Em SVs pequenos, Ktrans foi menor (0,13 vs 0,029, *p* < 0,001), Kep foi maior (0,68 vs 0,46, *p* = 0,037) e Ve foi menor (0,45 vs 0,83, *p* < 0,001) que nos SVs grandes. Ktrans e Ve foram menores dentro dos cistos que nas porções solidas dos SVs císticos (0,14 vs 0,32, *p* < 0,001; 0,37 vs 0,78, *p* < 0.001, respectivamente). Foi encontrada correlação positiva em Ktrans (r = 0,71, *p* = 0,048) e Kep (r = 0,74, *p* = 0,037) entre as áreas sólidas e císticas.

**Conclusão:**

Nos SVs, o tamanho está consistentemente associado com os valores da perfusão. Nos SVs císticos, as porções císticas parecem ter valores menores de Ktrans e Ve do que nas porções sólidas.

## INTRODUCTION

Perfusion-weighted imaging (PWI) has revolutionized the field of neurological oncology, improving the differential diagnosis and grading of gliomas and other intraparenchymal tumors^(1,2,3,4,5,6)^, although its impact has been less pronounced for extra-axial lesions. For skull base tumors such as vestibular schwannoma (VS) and meningioma, the typical characteristics on standard computed tomography and magnetic resonance imaging (MRI) are well known; therefore, PWI has not been necessary for their diagnosis^(7)^. Studies of VS have been focused almost exclusively on the differential diagnosis with intraparenchymal lesions and doing so by considering VS as a single entity, which seems counterintuitive, given that the heterogeneity of signal intensity and contrast uptake dynamics in VS have been well described^(1,8,9)^. In addition, researchers have excluded small VSs, because of artifacts around the temporal bone; hence, it is not possible to know if previous results can be extrapolated to all VSs. Here, we present a pilot study to assess the viability of PWI in VS, regardless of tumor size, as well as to describe the effects that the size of the tumor and its structure (solid, cystic, or heterogeneous) have on perfusion values.

## MATERIALS AND METHODS

### Subjects

Between August 2018 and August 2019, a total of 48 patients with VS underwent surgical resection at the Neurological Institute of Curitiba, Brazil. Of those 48 patients, 20 had untreated, sporadic VS and were selected for analysis. All of those patients were referred for surgery at the time of diagnosis; therefore, there were no cases for which there were previous imaging studies that would have allowed us to assess growth. In all cases, the patients underwent preoperative PWI, the histological analysis resulted in a diagnosis of schwannoma, and that diagnosis was confirmed intraoperatively. All of the schwannomas were found to have arisen from one of the branches of the vestibular nerve. The study was approved by the local institutional review board. Because of the observational nature of the study, the requirement to obtain informed consent was waived.

### Image acquisition

All MRI scans were acquired in a 1.5-T scanner (Signa HDxt; General Electric, Milwaukee, WI, USA), with an 8-channel head coil. The protocol consisted of T1-weighted, T2-weighted, diffusion-weighted, T2*-weighted, and contrast-enhanced T1-weighted three-dimensional fast spoiled gradient echo images. High-resolution three-dimensional fast imaging employing steady-state acquisition (FIESTA) sequences were acquired—repetition time (TR) = 4,900 ms; echo time (TE) = 1,800 ms; flip angle = 60°; slice thickness = 0.8 mm; acquisition time = 3 min 40 s—as were diffusion-weighted images with automatic apparent diffusion coefficient mapping—TR = 9,000 ms; TE = 99 ms; flip angle = 90°; b value = 1,000; thickness = 6 mm; acquisition time = 1 min 20 s. Dynamic contrast-enhanced (DCE) perfusion was performed by using T1-weighted sequences—TR = minimum; TE = minimum; 44 phases, 7 s apart; flip angle = 25°; field of view = 28 mm; slice thickness = 6 mm; acquisition time = 5 min 13 s. A bolus of gadodiamide (Omniscan, 0.1 mmol; GE Healthcare) was injected at a rate of 2 mL/s. That also served to decrease the T1 effect before a second dose of gadodiamide (0.1 mmol/kg) was injected (at 4 mL/s) for dynamic susceptibility contrast (DSC) perfusion MRI to determine the relative cerebral blood volume (rCBV). The DSC imaging was performed by using T2*-weighted echo-planar imaging gradient-echo sequence—TR = 1,200 ms; TE = 50 ms; flip angle = 60°; slice thickness = 8 mm; acquisition time = 1 min 24 s. Because of the slice thickness, a neuroradiologist supervised the image acquisition in order to center the images over the tumor and ensure proper quality, especially for small lesions.

### Image analysis

**Figure 1:**
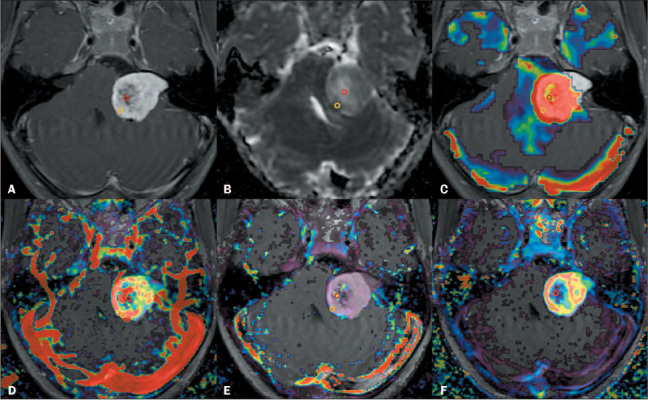
MRI scans of a 41-year- old female with a left-sided Hannover T4 cystic VS. Images show the positioning of ROIs in the solid portions (yellow circles) and in the cystic portions (red circles). A: Axial contrast-enhanced T1-weighted image. B: Apparent diffusion coefficient map. C: Fused DSC-MRI/PWI scan showing the rCBV. D: Fused DCE-MRI/PWI scan showing the Ktrans. E: Fused DCE-MRI/PWI scan showing the Kep. F: Fused DCE-MRI/PWI scan showing the Ve. Note the strong contrast enhancement in the solid portions (yellow circles) and the weak contrast enhancement in the cystic portions (red circles). Note also that there are considerably fewer susceptibility artifacts on DCE-MRI (D–F) than on DSC-MRI (C).

**Figure 2:**
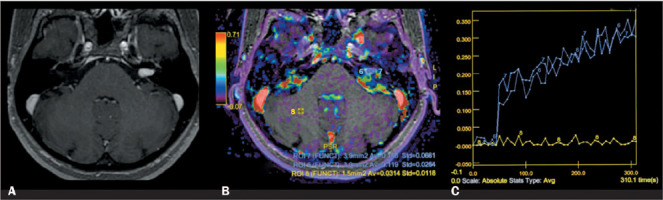
MRI scans of a 64-year-old female with a left-sided Hannover T2 VS. A: Axial contrast- enhanced T1-weighted image. B: Fused perfusion map with a contrast-enhanced image showing ROIs over the tumor (numbers 6 and 7) and cerebellum (number 8). C: Time-signal intensity curves for both ROIs over the tumor (blue lines) compared with that for the ROI over the cerebellum (yellow line).

Images were registered and fused by using the integrated registration application of an Advantage Workstation 4.7 (GE Medical Systems, Milwaukee, WI, USA), in which colored multiparametric perfusion maps were automatically generated with GenIQ software (GE Medical Systems) for DCE analyses and with BrainStat AIF software (GE Medical Systems) for DSC analyses. Small (5-mm^2^) regions of interest (ROIs) were independently placed at the site of highest perfusion value (i.e., the hot spot method was employed) by two neuroradiologists with 10 and 30 years of experience, respectively. In two cases, there was a discrepancy in ROI placement between the two neuroradiologists, which was resolved by consensus. The rCBV values, as well as those for parameters related to the extravascular extracellular space (EES)—the volume transfer constant between a vessel and the EES (Ktrans, which could be simplified as the rate at which contrast enters the tumor), the EES volume per unit of tissue volume (Ve, which could be interpreted as the quantity of extracellular space in that area), and the rate transfer constant between EES and blood plasma (Kep, which could be interpreted as the rate at which contrast leaks out of the tumor)—were calculated automatically (Figures 1 and 2).

Tumors were rated according to the Hannover classification^(10)^, as follows: T1, for purely intracanalicular tumors; T2, for tumors with a small cisternal extension; T3, for tumors that filled the cerebellopontine angle cistern without reaching the brainstem; or T4, for tumors that were in contact with the brainstem. Hannover T1 and T2 tumors were categorized as small VSs, whereas Hannover T3 and T4 tumors were categorized as large VSs. We also categorized tumors as described by Constanzo et al.^(11)^: solid, if they showed homogeneous contrast enhancement; heterogeneous, if they contained areas without contrast enhancement, but no cystic cavities were seen on a FIESTA sequence; or cystic, if a FIESTA sequence showed that they contained hyperintense cavities without contrast enhancement. Heterogeneous tumors were categorized as cystic VSs for statistical analysis, on the basis of the hypothesis that such tumors are in the initial stage of cystic degeneration^(11)^. For each cystic VS, an additional ROI was placed over the center of the largest cyst. Statistical analysis of cystic VSs was performed using the value for the hot-spot (solid portion), unless the value for the cystic portion was explicitly stated.

### Statistical analysis

For the statistical analysis, we used unpaired Student’s t-tests and Spearman’s rank correlation coefficient to detect linear associations. Values of *p* ≤ 0.05 were considered statistically significant.

## RESULTS

Twenty patients were included in the analysis. The mean age was 47 years (range, 18–78 years). The male-to-female ratio was 1:1. There were two patients with Hannover T1 tumors, three with Hannover T2 tumors, one with a Hannover T3 tumor, and 14 with Hannover T4 tumors. Of the 20 VSs evaluated, 10 (two Hannover T1 tumors, two Hannover T2 tumors, one Hannover T3 tumor, and five Hannover T4 tumors) were categorized as solid, two (one Hannover T2 tumor and one Hannover T3 tumor) were categorized as heterogeneous, and eight (all Hannover T4 tumors) were categorized as cystic.

### Perfusion values

The DCE-MRI-derived parameters (Ktrans, Ve, and Kep) were measurable in all cases, whereas artifacts related to the bone–air interfaces in the temporal bone prevented us from obtaining rCBV values in eight VSs (two solid Hannover T1 tumors, two solid Hannover T2 tumors, one heterogeneous Hannover T2 tumor, one heterogeneous Hannover T3 tumor, and two cystic Hannover T4 tumors). There was a moderate positive correlation between Ktrans and rCBV (Table 1).

**Table 1:** Spearman’s rank correlation coefficient between perfusion values.

Category	Kep	*P*	Ve	*P*	rCBV	*P*
All VSs						
Ktrans	0.03	0.886	0.42	0.062	0.62	0.031
Kep	—	—	–0.51	0.021	0.55	0.062
Ve	—	—	—	—	–0.33	0.296
Small VSs						
Ktrans	0.1	0.873	0.8	0.104	—	—
Kep	—	—	0.1	0.873	—	—
Large VSs						
Ktrans	0.53	0.041	–0.18	0.513	0.62	0.031
Kep	—	—	–0.38	0.161	0.55	0.062
Ve	—	—	—	—	–0.33	0.296

### Hannover classification

The mean values for Ktrans and Ve were lower in the small tumors than in the large tumors, whereas the mean Kep value was higher in the former (Table 2). As shown in Table 1, there was a moderate correlation between Ktrans and rCBV in the large VSs (it was not possible to determine the rCBV in the small VSs).

**Table 2:** Differences in perfusion values, by VS size.

Size	Ktrans Mean ± SD	*P* (95% CI)	Kep Mean ± SD	*P* (95% CI)	Ve Mean ± SD	*P* (95% CI)
Small	0.13 (0.06)	< 0.001 (–0.24 to –0.08)	0.68 (0.20)	0.037 (0.01 to 0.43)	0.45 (0.15)	< 0.001 (–0.53 to –0.23)
Large	0.29 (0.08)		0.46 (0.19)		0.83 (0.14)	
T1	0.08 (0.04)	0.007 (–0.33 to –0.06)	0.62 (0.37)	0.45 (–0.21 to 0.45)	0.37 (0.05)	0.007 (–0.70 to –0.13)
All others	0.27 (0.09)		0.50 (0.20)		0.78 (0.19)	
T4	0.30 (0.08)	0.002 (0.06 to 0.22)	0.46 (0.19)	0.09 (–0.38 to 0.03)	0.84 (0.14)	< 0.001 (0.17 to 0.49)
All others	0.16 (0.08)		0.63 (0.21)		0.51 (0.19)	

SD, standard deviation; 95% CI, 95% confidence interval.

### Cystic classification

The perfusion “hot spot” corresponded to the solid component of heterogeneous and cystic VSs. The Ktrans was lowest in the solid tumors and highest in the cystic tumors, although there was no difference between the large solid tumors and the large cystic tumors in terms of the perfusion values. Among the solid VSs, the Ktrans and Ve were higher in those categorized as large (Table 3) and there was a moderate correlation between Ktrans and Ve (Table 4).

**Table 3:** Differences in perfusion values, by size and cystic status.

Category	Ktrans Mean ± SD	*P* (95% CI)	Kep Mean ± SD	*P* (95% CI)	Ve Mean ± SD	*P* (95% CI)	rCBV Mean ± SD	*P* (95% CI)
All VSs								
Solid	0.21 (0.10)	0.029 (–0.18 to –0.01)	0.49 (0.23)	0.688 (–0.24 to 0.16)	0.72 (0.25)	0.722 (–0.25 to 0.17)	7.75 (5.57)	0.438 (–7.7 to 3.6)
Cystic	0.30 (0.08)		0.53 (0.20)		0.76 (0.19)		9.8 (2.75)	
Large VSs								
Solid	0.27 (0.08)	0.3 (–0.13 to 0.04)	0.37 (0.15)	0.175 (–0.34 to 0.07)	0.90 (0.06)	0.152 (–0.45 to 0.26)	7.75 (5.57)	0.438 (–7.7 to 3.6)
Cystic	0.31 (0.08)		0.51 (0.20)		0.79 (0.16)		9.8 (2.75)	
Solid VSs								
Small	0.11 (0.05)	0.009 (–0.26 to –0.05)	0.67 (0.23)	0.039 (0.02 to 0.57)	0.46 (0.17)	< 0.001 (–0.61 to –0.27)	—	—
Large	0.27 (0.08)		0.37 (0.15)		0.90 (0.06)		—	
Cystic VSs								
Solid Part	0.32 (0.08)	< 0.001 (0.11 to 0.25)	0.53 (0.20)	0.927 (–0.39 to 0.36)	0.78 (0.17)	< 0.001 (0.24 to 0.57)	9.8 (2.75)	0.541 (–9.7 to 5.5)
Cystic Part	0.14 (0.04)		0.55 (0.45)		0.37 (0.14)		11.87 (7.32)	

SD, standard deviation; 95% CI, 95% confidence interval.

**Table 4:** Spearman’s rank correlation coefficients between perfusion values in solid VSs.

Category	Kep	*P*	Ve	*P*	rCBV	*P*
All solid VSs						
Ktrans	–0.21	0.555	0.64	0.048	0.60	0.208
Kep	—	—	–0.52	0.121	0.72	0.103
Ve	—	—	—	—	–0.60	0.208
Large solid VSs						
Ktrans	0.67	0.148	–0.66	0.156	0.6	0.208
Kep	—	—	–0.41	0.425	0.72	0.103
Ve	—	—	—	—	–0.6	0.208

In cystic VSs, the Ktrans and Ve values were lower in the cystic portions than in the solid portions (Table 3). There were significant positive correlations between the solid and cystic portions for Ktrans (r = 0.71, *p* = 0.048) and Kep (r = 0.74, *p* = 0.037), although not for Ve (r = 0.70, *p* = 0.056). In the cystic VSs, there were no significant correlations between any of the PWI parameters (Table 5).

**Table 5:** Differences between perfusion values in the solid and cystic portions of cystic VSs.

Cystic VS portion	Kep	*P*	Ve	*P*	rCBV	*P*
Solid						
Ktrans	0.31	0.456	0.10	0.823	0.54	0.266
Kep	—	—	–0.55	0.160	0.71	0.111
Ve	—	—	—	—	–0.14	0.787
Cystic						
Ktrans	0.66	0.073	–0.35	0.402	—	—
Kep	—	—	–0.49	0.217	—	—
Ve	—	—	—	—	—	—

## DISCUSSION

Our results show that small VSs can be evaluated with DCE-MRI but not with DSC-MRI. We detected a direct correlation between Ktrans and rCBV (measurements of permeability and perfusion, respectively) and an indirect correlation between Ve and Kep (a larger EES results in a lower transfer coefficient between the EES and plasma). We also found that the Ktrans and Ve were greater in larger tumors than in small tumors, whereas the Kep was lower in the larger tumors. We observed no differences in perfusion values between the solid tumors and the heterogeneous/cystic tumors of similar size. However, the Ktrans and Ve were lower in the cystic portions than in the solid portions.

There are a number of limitations to the use of PWI, which have precluded its widespread adoption into the standard evaluation of skull base tumors. Such limitations include the lack of clinical significance, the fact that the tumors are located near bone–air interfaces, and the typically small size of the tumors. The few studies that have included schwannomas in their analyses have considered them as a single entity^(1,3,8,9,12,13,14)^, failing to take into account the heterogeneous contrast uptake frequently seen on MRI and excluding small tumors.

Regarding size, DCE-MRI has the theoretical advantage of better spatial resolution and less susceptibility artifacts around bone–air interfaces (e.g., the mastoid bone) in comparison with DSC-MRI^(8,15,16,17,18)^. We were able to confirm this, showing that DCE-MRI allowed proper ROI placement in small intracanalicular VSs, obtaining similar values after placing several ROIs in contiguous locations and perfusion curves consistent with those obtained in larger VSs (Figure 2). Therefore, there is no evidence that artifacts interfered with our measurements in small VSs.

In general terms, DCE-derived quantitative parameters reflect microcirculatory structure and function^(19)^, Ktrans correlating with vascularity markers such as vascular endothelial growth factor^(4)^ and Ve correlating with histological estimates of EES volume^(5,6,18)^. The Ktrans depends on vascular surface area and flow^(20,21)^. Therefore, when there is high permeability, the influx of contrast is limited only by flow, whereas low permeability impedes the passage of contrast into the EES^(22)^. This is further complicated in schwannomas, given that histological studies have demonstrated high permeability due to the lack of a blood–brain barrier within the tumor^(23)^, as well as to the presence of arteriovenous shunts producing high intratumoral flow, without contributing to perfusion^(17,24)^. Consequently, within a large VS, areas of low and high perfusion may coexist, which is the basis for the idea that perfusion is not homogeneous within a VS. By considering VS as a single entity, previous authors concluded that the EES is larger in VSs than in meningiomas and gliomas^(1,12)^, as well as that rCBV and total blood flow are lower^(1,14)^. However, we found that perfusion values varied according to tumor size and even between different areas of the same tumor, demonstrating how illogical it is to group schwannomas together as a single entity. In fact, because any solid tumor with a volume > 2mm^3^ requires angiogenesis for maintenance^(25)^, we expected the Ktrans values to be higher for larger tumors than for smaller tumors. That was also reported by Lewis et al.^(18)^, although those authors excluded small VSs. Similarly, we detected a positive correlation between permeability and blood volume, which has been described in cases of glioma but not in cases of VS^(3)^. That correlation was lost in the small tumors and was not significant within the cystic portions of the larger tumors, indicating that permeability and perfusion were not linked in the cystic portions, as they were in the solid portions. Zhu et al.^(1)^ were the first to describe this intralesional variability. These observations support the notion that some large VSs have areas of high flow and low permeability (arteriovenous shunts) within the highly permeable tumor. In addition, it seems that this decoupling is associated with cystic degeneration, given that our data show that the mean Ktrans was 56% lower in the cystic portion than in the solid portion, whereas the mean rCBV was 21% higher in the cystic portion.

Although some authors have stated that VSs always become cystic when they grow larger than 25 mm^(26)^, other authors have reported different biologic behavior^(27)^. Although cystic degeneration is visible only in large tumors, large solid VSs are still more common than are large cystic ones^(27,28)^. Whereas small VSs contain exclusively compact, cellular tissue (known as Antoni A areas), large VSs contain Antoni A areas mixed with loosely arranged cells with microcystic spaces filled with mucin (known as Antoni B areas)^(26)^. Some authors postulate that Antoni B areas coalesce to create large cysts^(29)^, although others have found microcystic elements in small tumors (those with Antoni A areas only), suggesting that all VSs have the potential for cystic degeneration^(30)^. Our results are in agreement with the latter, given that we found no difference in the Ktrans observed for large solid VSs and that observed for the solid portions of all cystic VSs. Macrocystic elements have been ascribed to fluid accumulation caused by an osmotic effect and to the extravasation of serum proteins resulting from an impaired blood–brain barrier^(31)^, although other authors have ascribed them to hemorrhage and mucinous degeneration^(32,33)^. Our data are in line with the second hypothesis, because the positive correlation between tumor size and EES volume was lost in cystic VSs, the mean Ve being 53% lower in the cystic portions than in the solid portions. In previous studies, the non-enhancing (cystic) portions of VSs have been shown to decrease significantly over time, with slow contrast uptake in up to 90% of the cystic portions^(34)^, further confirming that cystic portions have different contrast uptake dynamics and that the cysts are cellular rather than liquid cavities, which explains the differences we found between the cystic and solid portions of cystic VSs in terms of perfusion and EES volume.

**Figure 3:**
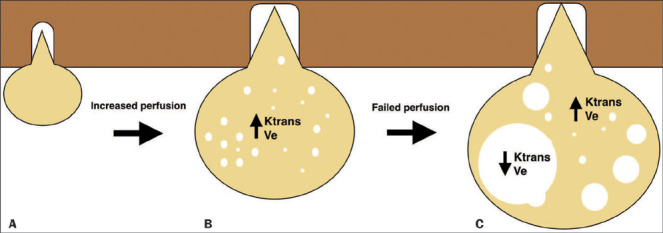
Hypothesis to explain failed perfusion. Growth of a small VS (A) into a large one (B) requires angiogenesis, which increases Ktrans and Ve. After a significant period of time, perfusion is unable to maintain growth in some areas of the tumor, and microcystic elements coalesce to produce radiologically visible cysts with decreased Ktrans and Ve, both of which remain high in the solid portions (C).

In summary, our results are consistent with the known biological behavior of VSs. Our data also shed further light on the possible pathophysiological relationship between perfusion and cystic degeneration: as schwannomas grow, Ktrans, rCBV, and Ve increase proportionally, either until perfusion of some parts of the tumor cannot keep pace with growth and macrocystic degeneration begins or until microcysts coalesce to form macrocysts, thus altering permeability (decreasing Ktrans, increasing rCBV, and decreasing Ve) in those areas. If we take into consideration the fact that arteriovenous shunts can cause high flow/low permeability, leading to ineffective blood supply in the region^(35)^, the first hypothesis (that some parts of the tumor cannot keep pace with growth and macrocystic degeneration begins) seems more plausible. However, both hypotheses would ultimately uncouple the association between blood flow and permeability (Figure 3).

### Limitations and future directions

Previous studies analyzing perfusion in VS actively excluded small lesions, thus failing to evaluate the influence that tumor size has on PWI values. To our knowledge, this is the first report including intracanalicular VSs and demonstrating that DCE-MRI is a feasible method for analyzing small skull base lesions. Even though the number of cases was small, we were able to obtain significant results that are, notably, in accordance with data in the literature and with the known biological behavior of VSs. The main obstacle to obtaining such results was the necessity of having a neuroradiologist present to ensure that the image acquisition was appropriate. Another limitation of our study was the lack of imaging follow-up, due to all patients having been selected from a surgical database. Serial imaging could have confirmed our hypothesis of perfusion failure and would have allowed us to identify the areas within a tumor that would evolve into a cyst.

It is important to recognize the clinical role PWI may play in the follow up of VS. Kleijwegt et al.^(16)^ used DSC-MRI to predict the growth of VS, and Li et al.^(36)^ used DCE-MRI to predict the response to bevacizumab in neurofibromatosis type 2-associated VS. Although those studies carry the promise of a change in the paradigm of VS management, they, like other studies, excluded small tumors and did not consider cystic status. Nevertheless, they showed that PWI can be clinically useful in VS, as also evidenced by our findings supporting the relationship between tumor size and perfusion values.

Despite the small size of our study sample, our findings are in keeping with data in the literature on the clinical benefit of PWI in VS. We showed that VS cannot be considered to have a single perfusion profile. Therefore, comparisons with other tumors must take size and cystic status into account, which could invalidate the results of some previous studies. In addition, given the heterogeneity of PWI within VS, we could also suggest further studies focusing on the long-term follow-up of VS and segmental evaluation of areas of cystic degeneration before they appear, as well as on the correlation between PWI and vestibulocochlear testing. We also believe that PWI can be particularly useful in the follow-up of intracanalicular schwannomas, because, given the inflammatory hypothesis previously stated, increased perfusion values might be associated not only with growth but also with hearing loss.

Finally, we must address the inherent bias of the “hot spot” method. Because it is based on a qualitative evaluation of the perfusion map, placing the ROI in another location will give a different result. However, that also indicates that there are different permeability profiles within a given VS. We fused contrast-enhanced images with those obtained by PWI in order to enhance the tumor margins and reduce selection bias, which allowed us to select the maximal values within the lesion. Therefore, we were able to use a single, unified variable for analysis, although further studies involving segmented evaluation and histogram analysis could also produce interesting results.

## CONCLUSION

Because VSs are heterogeneous lesions, they can present different perfusion profiles within the tumor. Small VSs can be evaluated accurately with DCE-MRI. Tumor size appears to be the most important factor influencing perfusion values. Within cystic VSs, permeability and EES volume seem to be lower in the cystic portions than in the solid portions.
